# Cardiac mechanics and incident ischemic stroke: the Cardiovascular Health Study

**DOI:** 10.1038/s41598-021-96702-z

**Published:** 2021-08-30

**Authors:** Hooman Kamel, Traci M. Bartz, W. T. Longstreth, Mitchell S. V. Elkind, John Gottdiener, Jorge R. Kizer, Julius M. Gardin, Jiwon Kim, Sanjiv Shah

**Affiliations:** 1 Clinical and Translational Neuroscience Unit, Department of Neurology and Feil Family Brain and Mind Research Institute, New York, NY USA; 2 Division of Neurocritical Care, Weill Cornell Medicine, 420 East 70th St, LH-413, New York, NY 10021 USA; 3 Departments of Biostatistics, University of Washington, Seattle, WA USA; 4 Departments of Neurology, University of Washington, Seattle, WA USA; 5 Departments of Medicine, University of Washington, Seattle, WA USA; 6 Departments of Epidemiology, University of Washington, Seattle, WA USA; 7 Department of Neurology, Vagelos College of Physicians and Surgeons, New York, USA; 8 Department of Epidemiology, Mailman School of Public Health, Columbia University, New York, NY USA; 9 Division of Cardiology, University of Maryland, Baltimore, MD USA; 10 Cardiology Section, San Francisco Veterans Affairs Health Care System, and Departments of Medicine, Epidemiology, and Biostatistics, University of California San Francisco, San Francisco, CA USA; 11 Division of Cardiology, Rutgers New Jersey Medical School, Newark, NJ USA; 12 Division of Cardiology, Weill Cornell Medicine, New York, NY USA; 13 Division of Cardiology, Northwestern University Feinberg School of Medicine, Chicago, IL USA

**Keywords:** Neurology, Cerebrovascular disorders, Stroke, Cardiology, Arrhythmias

## Abstract

Recent evidence indicates that our understanding of the relationship between cardiac function and ischemic stroke remains incomplete. The Cardiovascular Health Study enrolled community-dwelling adults ≥ 65 years old. We included participants with speckle-tracking data from digitized baseline study echocardiograms. Exposures were left atrial reservoir strain (primary), left ventricular longitudinal strain, left ventricular early diastolic strain rate, septal e’ velocity, and lateral e’ velocity. The primary outcome was incident ischemic stroke. Cox proportional hazards models were adjusted for demographics, image quality, and risk factors including left ventricular ejection fraction and incident atrial fibrillation. Among 4,000 participants in our analysis, lower (worse) left atrial reservoir strain was associated with incident ischemic stroke (HR per SD absolute decrease, 1.14; 95% CI 1.04–25). All secondary exposure variables were significantly associated with the outcome. Left atrial reservoir strain was associated with cardioembolic stroke (HR per SD absolute decrease, 1.42; 95% CI 1.21–1.67) and cardioembolic stroke related to incident atrial fibrillation (HR per SD absolute decrease, 1.60; 1.32–1.95). Myocardial dysfunction that can ultimately lead to stroke may be identifiable at an early stage. This highlights opportunities to identify cerebrovascular risk earlier and improve stroke prevention via therapies for early myocardial dysfunction.

Strokes account for 10% of deaths worldwide^1^, and two-thirds of strokes are ischemic strokes. A substantial and increasing proportion of ischemic strokes result from cardiac embolism^2^. Cardioembolic strokes cause more disability than other types of ischemic stroke^3^, making cardiac disease an especially important target for stroke prevention efforts. Several cardiac conditions have been firmly established as stroke risk factors, most notably atrial fibrillation (AF), heart failure with reduced ejection fraction, acute myocardial infarction (MI), and severe valvular disease^4^. Recent studies suggest that our understanding of the relationships between cardiac structure and function and ischemic stroke remains incomplete^4–8^. At the same time, approximately one-fifth of ischemic strokes do not have a specific identifiable cause^9^, and most of these cryptogenic strokes are likely caused by emboli from currently unrecognized sources in the heart, aorta, or large cerebral vessels^10^. An improved understanding of the relationship between cardiac disease and ischemic stroke may thus shed light on currently unrecognized sources of stroke and lead to better therapy for stroke prevention.

Speckle tracking is a validated technique that allows for quantification of myocardial tissue deformation using two-dimensional echocardiographic images^11,12^. With this technique, subtle cardiac dysfunction can be detected before the appearance of overt structural abnormalities or clinical manifestations^13^. Myocardial strain indices have been associated with incident AF^14–17^, markers of left atrial thromboembolism^18,19^, and stroke in patients with AF^20,21^ or acute MI. Few data exist on the association between myocardial strain and incident ischemic stroke in the general population. Since speckle-tracking echocardiography illuminates aspects of cardiac function that are not readily detectable by conventional echocardiography, elucidating the relationship between myocardial strain and stroke may fill gaps in knowledge about cardioembolic stroke. Using data from participants in a prospective, longitudinal cohort study, we tested the hypothesis that indices of cardiac mechanics, especially those related to atrial function, are independently associated with subsequent ischemic stroke.

## Methods

### Design

The Cardiovascular Health Study (CHS) prospectively enrolled and follows a community-dwelling cohort of individuals ≥ 65 years of age. CHS study centers recruited a first cohort of 5,201 participants in 1989–1990 and a second, predominantly African-American, cohort of 687 participants in 1992–1993. These 5,888 participants were selected from a random sample of people on Medicare eligibility lists in four counties, one each in California, Maryland, North Carolina, and Pennsylvania^22^. Participants returned for in-person study visits annually until 1998–1999 and again in 2005–2006. Throughout follow-up, participants were contacted via semiannual telephone calls to ascertain events, and follow-up data were also linked with Medicare claims. Institutional review boards at the University of Washington and each field center approved this study, and all participants provided written informed consent. All methods were performed in accordance with the relevant guidelines and regulations.

### Participants

CHS excluded individuals who were younger than 65 years of age, could not give consent or answer questions without a surrogate, resided in an institutional setting, were wheelchair dependent, or were receiving active treatment for cancer. For this study, we included participants with available speckle-tracking data from their baseline echocardiogram. We excluded participants who had experienced a stroke (ischemic or hemorrhagic) or were diagnosed with AF at or prior to their baseline echocardiogram.

### Measurements

Comprehensive 2-dimensional, M-mode, and Doppler echocardiograms were obtained in 1989–1990 and again in 1994–1995, and they served as the baseline scans for the original and supplemental cohorts, respectively^23^. The echocardiograms were performed using a standardized protocol and were interpreted at core reading centers. At the four field centers, echocardiograms were recorded onto Super VHS tapes using Toshiba SSH-160A cardiac ultrasound machines. Videotapes were sent to the CHS Echocardiography Reading Center (Irvine, CA, USA, for the 1989–1990 echocardiograms, and Washington, DC, USA for the 1994–1995 echocardiograms), where images were digitized and initial measurements were made using M-mode, 2-D, and Doppler images. From 2016 to 2018, archived CHS echocardiograms were digitized using the TIMS 2000 DICOM system (Foresight Imaging, Chelmsford, Massachusetts, USA), using methods developed by our group for a similar analysis done in the Hypertension Genetic Epidemiology Study (HyperGEN Study)^13^. Cine loops of 2–4 cardiac cycles from the apical 4-chamber^24^ view were digitized at a frame rate of 30 frames per second and stored offline in DICOM format (Northwestern University, Chicago, IL, USA). Digitized cine loops of the apical 4-chamber view from the highest-quality cardiac cycle were analyzed using 2-dimensional wall-motion tracking software (2D Cardiac Performance Analysis, TomTec v4.5, Unterschleisshein, Germany).

Our echocardiographic exposure variables were prespecified based on an evolving conceptual understanding of the relationship between myocardial disease and thromboembolism, which posits a central role for a thrombogenic atrial myopathy resulting from ventricular dysfunction, primary atrial dysfunction, and/or systemic inflammation^4–8,25–28^. The primary exposure variable was left atrial reservoir strain, which is a hallmark of atrial myopathy and represents the degree of deformation as the left atrium fills and stretches during ventricular systole^26^. Secondary exposure variables were related to measures of left ventricular function: left ventricular average longitudinal strain, early diastolic strain rate, septal e’ velocity, and lateral e’ velocity.

For both the left ventricle and left atrium, 6 segmental strain curves were measured for each chamber in the apical 4-chamber view using electrocardiographic R-to-R wave gating. Speckle-tracking analysis was only performed in the apical 4-chamber view and not in all 3 apical views for feasibility purposes and based on our prior finding that, in population-based studies, strain parameters from all 3 apical views are highly correlated^13^. For left ventricular longitudinal strain, average longitudinal strain was the average of the 6 segmental strain curves at ventricular end-systole. Early diastolic (e’) velocities measured by speckle-tracking are lower than tissue Doppler velocities used clinically because they represent the average and not peak e’ velocity at the septal and lateral mitral annulus^13^. Experienced operators blinded to all other variables performed the speckle-tracking analysis and assessed chamber-specific tracking quality using a standard 4-point scale^13^. This method of measuring cardiac mechanics from archived echocardiograms has been validated against native digital speckle tracking on modern echocardiography machines^13^. Inter- and intra-observer reproducibility demonstrated high reliability and relatively low bias (Online Table I).

The primary outcome was incident ischemic stroke. Methods for identifying and adjudicating strokes in CHS have been previously published^29^. Stroke was defined as the rapid onset of a neurological deficit lasting > 24 h or until death, or a lesion on computed tomography or magnetic resonance imaging if symptoms lasted < 24 h, and no evidence that the symptoms were due to brain trauma, tumor, or infection. To be classified as an ischemic stroke, a stroke also had to involve: (1) a focal neurological deficit without evidence of intracranial hemorrhage on computed tomography, magnetic resonance imaging, or cerebrospinal fluid analysis, or (2) imaging evidence of brain ischemia in a location compatible with the presenting symptoms.

Potential confounders included in multivariable models were age, sex, race (African-American versus other race), education level (< high school level versus high school or more), body mass index, coronary heart disease, heart failure, diabetes, anti-hypertensive medications and systolic blood pressure, high- and low-density lipoprotein and triglyceride levels, smoking status (never, past, or current), left ventricular ejection fraction (≥ 55%, 45–54%, or < 45%), CHS site, speckle-tracking analyst, chamber-specific echocardiogram image quality (using a standard 4-point scale^13^), and a time-varying variable for incident AF during follow-up. Cholesterol and triglyceride levels were from 2 years prior to the baseline echocardiogram for the second cohort; all other covariates were defined at the time of the baseline echocardiogram. Incident AF was defined through 2014 from study visit ECGs, hospital discharge diagnoses including those ascertained from inpatient Medicare claims, and diagnoses from outpatient or physician service claims from Medicare data. AF occurring only during a hospitalization for valve surgery or coronary artery bypass grafting was not considered an AF event.

### Statistical analysis

Due to the timing of echocardiographic assessments, the baseline for analyses was 1989–1990 for the first (original) cohort and 1994–1995 (2 years after study baseline) for the second cohort. Baseline data were summarized separately for CHS participants with and without speckle-tracking echocardiography data who otherwise met inclusion criteria for analysis. Baseline characteristics were reported as mean and standard deviation (SD) for continuous variables and number and percent for categorical variables. Strain and tissue velocity measurements were converted to absolute values, with lower absolute values representing worse strain and tissue velocities.

After verification of the proportional hazards assumption, Cox proportional hazards analysis was used to model the association between the exposure variables and incident ischemic stroke. Participants were censored at the time of hemorrhagic stroke or stroke of unknown type, last follow-up, or administrative censoring (June 31, 2015) or death if they occurred prior to ischemic stroke. We determined the functional form of exposure variables with penalized cubic splines before including these variables in the Cox model. As the associations were reasonably linear, we modeled exposure variables without transformation or the use of splines in the following main models. Model 1 was adjusted for age, race, sex, CHS site, speckle-tracking analyst, and chamber-specific image quality. Model 2 was additionally adjusted for education, body mass index, coronary heart disease, heart failure, diabetes, systolic blood pressure, anti-hypertension medication, high-density lipoprotein level, low-density lipoprotein level, triglyceride level, smoking status, and left ventricular ejection fraction. Model 3 was additionally adjusted for AF as a time-varying covariate. Model 4 was applied only to our secondary exposure variables, and, in addition to the variables in Model 2, was also adjusted for our primary exposure variable of left atrial reservoir strain as well as left atrial image quality, with the goal of exploring whether any relationships between left ventricular mechanics and stroke were mediated by left atrial mechanics.

We performed several sensitivity analyses. First, we included only participants in the first cohort who underwent echocardiography at the same time as the study baseline. Second, we included participants with prevalent AF and instead adjusted for this baseline covariate. Third, we excluded participants with prevalent coronary heart disease or heart failure. Fourth, we additionally adjusted for left atrial volume, which was measured on 2-dimensional echocardiogram images by tracing the left atrium at ventricular end-systole in the apical 4-chamber view, with the method of discs used to calculate left atrial volume. Fifth, in an analysis of our primary exposure of left atrial reservoir strain, we additionally adjusted for our secondary exposure variables, with the goal of exploring whether the relationship between left atrial mechanics and stroke was mediated by left ventricular mechanics.

We also performed post-hoc exploratory analyses of associations of our exposure variables with the specific ischemic stroke subtypes of cardioembolic stroke and AF-related cardioembolic stroke. Cardioembolic stroke was adjudicated by the CHS adjudication committee using standard definitions^29^. We defined AF-related cardioembolic stroke as the occurrence of a cardioembolic stroke in a participant who also had a diagnosis of incident AF before or at the same time as the cardioembolic stroke. All statistical tests were 2-tailed and the threshold of statistical significance was set at alpha = 0.05. Statistical analyses were performed using Stata 12.1 (StataCorp, College Station, TX, USA).

## Results

Of the 5,888 participants in the overall CHS cohort, 275 were excluded because of prevalent stroke at baseline, 1,304 because of missing speckle-tracking echocardiogram data, 127 because of prevalent AF at baseline, and 182 because of other missing covariates, resulting in 4,000 participants who were eligible for our analysis (Online Figure I). Of the 1,304 participants with missing speckle-tracking data, 1,029 otherwise met our inclusion criteria. These 1,029 otherwise-eligible participants who were excluded because of missing speckle-tracking data had broadly similar baseline characteristics to the 4,000 included participants except for race and location of recruitment (Table 1); these differences were due to the relative availability of echocardiogram data from the first versus second CHS cohorts. Among the 4,000 included participants, the mean left atrial reservoir strain was in the normal range^30^ (40.8% ± 15.4%; n = 3,892) and the mean left ventricular longitudinal strain was in the borderline range (16.7% ± 4.3%; n = 3,987) (Table 1)^31,32^.

**Table 1:** Baseline characteristics of cardiovascular health study participants*, stratified by availability of echocardiographic data on myocardial strain.

Characteristic^†^	Strain data available (N = 4,000)	Strain data missing (N = 1,029)
Age, mean (SD), years	73 (6)	73 (6)
Male	1,684 (42.1)	411 (39.9)
African-American	338 (8.5)	253 (24.6)
Did not complete high school	1,108 (27.7)	306 (29.7)
Body mass index, mean (SD)	26.3 (4.3)	27.6 (5.1)
Systolic blood pressure, mean (SD), mm Hg	136 (21)	138 (21)
High-density lipoprotein, mean (SD), mg/dL	55 (16)	55 (15)
Low-density lipoprotein, mean (SD), mg/dL	131 (36)	129 (35)
Triglycerides, mean (SD), mg/dL	135 (59)	135 (62)
Coronary heart disease	738 (18.4)	195 (19.0)
Heart failure	136 (3.4)	48 (4.7)
Diabetes	565 (14.1)	177 (17.2)
Antihypertension medication use	1,768 (44.2)	500 (48.6)
**Smoking status**		
Never	1,842 (46.1)	457 (44.4)
Former	1,691 (42.3)	450 (43.7)
Current	467 (11.7)	122 (11.9)
**Study recruitment site**		
Bowman Gray	1,038 (25.9)	284 (27.6)
Davis	987 (24.7)	265 (25.8)
Hopkins	1,007 (25.2)	155 (15.1)
Pittsburgh	968 (24.2)	325 (31.6)
**Left ventricular ejection fraction**		
Normal (≥ 55%)	3,669 (91.7)	940 (91.4)
Borderline (45–54%)	199 (5.0)	59 (5.7)
Abnormal (< 45%)	132 (3.3)	30 (2.9)
**Myocardial mechanics**^‡^		
LA reservoir strain, mean (SD), %	40.8 (15.4)	
LV average longitudinal strain, mean (SD), %	14.2 (3.8)	
LV early diastolic strain rate, mean (SD), s^−1^	0.65 (0.24)	
LV septal e’ velocity, mean (SD), cm/s^§^	2.94 (1.15)	
LV lateral e’ velocity, mean (SD), cm/s^§^	2.51 (1.19)	

LA, left atrial; LV, left ventricular; SD, standard deviation.

*Data shown are from the 5,029 of 5,888 Cardiovascular Health Study participants without prevalent stroke or atrial fibrillation who had available data on all other covariates at the time of their baseline echocardiogram.

^†^Data are presented as number (%) unless otherwise specified.

^‡^Strain measurements were converted to absolute values, with lower absolute values representing worse strain.

^§^Early diastolic (e’) velocities measured by speckle-tracking are lower than tissue Doppler velocities used clinically because they represent the average and not peak e’ velocity at the septal and lateral mitral annulus.

During a median 12.9 years of follow-up, 651 ischemic strokes occurred among the 4,000 CHS participants included in this analysis (16.3%). After adjustment for demographics and echocardiogram image quality, worse left atrial reservoir strain was associated with incident ischemic stroke (hazard ratio [HR] per standard deviation [SD] absolute decrease, 1.23; 95% confidence interval [CI] 1.13–1.34) (Table 2). This association was attenuated but persisted after further adjustment for left ventricular ejection fraction and vascular risk factors including a time-varying covariate for incident AF (HR per SD absolute decrease, 1.14; 95% CI 1.04–1.25). All secondary exposure variables were also significantly associated with incident ischemic stroke (Table 2). The associations between measures of left ventricular mechanics and stroke persisted after additional adjustment for left atrial reservoir strain (Table 2, Model 4); similarly, the association between left atrial reservoir strain and stroke persisted after adjustment for measures of left ventricular mechanics (Table 3, Sensitivity Analysis 5). The associations between measures of myocardial mechanics and ischemic stroke were not substantially different in sensitivity analyses that included only participants with echocardiograms at the time of study baseline, included participants with prevalent AF, excluded participants with prevalent coronary heart disease or heart failure, or additionally adjusted for left atrial volume (Table 3). Of the 651 ischemic strokes, 238 were classified as cardioembolic. In post-hoc exploratory analyses, all exposure variables with the exception of LV lateral e’ velocity were significantly associated with incident cardioembolic stroke (Table 4). In an analysis of 167 AF-related cardioembolic strokes, significant associations were present with left atrial reservoir strain and several secondary exposure variables (Table 4).

**Table 2:** Associations between myocardial mechanics and incident ischemic stroke in the cardiovascular health study.

Exposure variable*	Model 1^†^	Model 2^‡^	Model 3^§^	Model 4^||^
**Primary exposure variable**				
LA reservoir strain	1.23 (1.13–1.34)	1.17 (1.07–1.28)	1.14 (1.04–1.25)	
**Secondary exposure variables**				
LV average longitudinal strain	1.31 (1.20–1.43)	1.22 (1.12–1.34)	1.23 (1.12–1.34)	1.18 (1.08–1.30)
LV early diastolic strain rate	1.32 (1.20–1.44)	1.23 (1.12–1.34)	1.23 (1.13–1.35)	1.20 (1.10–1.32)
LV septal e’ velocity	1.24 (1.14–1.34)	1.17 (1.08–1.27)	1.18 (1.09–1.29)	1.16 (1.06–1.26)
LV lateral e’ velocity	1.13 (1.04–1.23)	1.09 (1.00–1.18)	1.09 (1.01–1.19)	1.09 (1.00–1.18)

LA, left atrial; LV, left ventricular.

*Reported results represent the hazard ratio (95% confidence interval) for ischemic stroke per each standard deviation absolute decrease in strain and decrease in strain rate and e’ velocity. Lower absolute values represent worse strain.

^†^Adjusted for age, race, sex, study site, speckle-tracking analyst, and chamber-specific echocardiogram image quality.

^‡^Adjusted for variables in Model 1 plus education, body mass index, coronary heart disease, heart failure, diabetes, systolic blood pressure, anti-hypertension medication, high-density lipoprotein level, low-density lipoprotein level, log-transformed triglyceride level, smoking status, and LV ejection fraction.

^§^Adjusted for variables in Model 2 plus atrial fibrillation as a time-varying covariate.

^||^Adjusted for variables in Model 2 plus LA reservoir strain and LA image quality score. The purpose of this model was to help assess whether the relationships between LV mechanics and stroke were mediated by LA mechanics.

**Table 3:** Sensitivity analyses of associations between myocardial mechanics and incident ischemic stroke in the cardiovascular health study.

Exposure variable*	Sensitivity analysis 1^†^	Sensitivity analysis 2^‡^	Sensitivity analysis 3^§^	Sensitivity analysis 4^||^	Sensitivity analysis 5^¶^
**Primary exposure variable**					
LA reservoir strain	1.16 (1.06–1.28)	1.14 (1.04–1.24)	1.17 (1.06–1.30)	1.13 (1.03–1.24)	1.11 (1.01–1.22)
**Secondary exposure variables**					
LV average longitudinal strain	1.23 (1.13–1.35)	1.22 (1.12–1.33)	1.22 (1.10–1.35)	1.23 (1.12–1.35)	
LV early diastolic strain rate	1.25 (1.14–1.37)	1.21 (1.11–1.32)	1.22 (1.11–1.35)	1.23 (1.12–1.35)	
LV septal e’ velocity	1.18 (1.09–1.29)	1.17 (1.08–1.27)	1.18 (1.07–1.29)	1.18 (1.08–1.28)	
LV lateral e’ velocity	1.12 (1.03–1.22)	1.08 (1.00–1.17)	1.11 (1.01–1.22)	1.10 (1.01–1.19)	

LA, left atrial; LV, left ventricular.

*Reported results represent the hazard ratio (95% confidence interval) for ischemic stroke per each standard deviation absolute decrease in strain and decrease in strain rate and e’ velocity. Lower absolute values represent worse strain. Reported models were adjusted for age, race, sex, study site, speckle-tracking analyst, chamber-specific echocardiogram image quality, education, body mass index, coronary heart disease, heart failure, diabetes, systolic blood pressure, anti-hypertension medication, high-density lipoprotein level, low-density lipoprotein level, log-transformed triglyceride level, smoking status, LV ejection fraction, and a time-varying covariate for atrial fibrillation.

^†^This sensitivity analysis included only participants with echocardiograms performed at study baseline.

^‡^This sensitivity analysis included participants with prevalent AF and instead adjusted for this baseline covariate.

^§^This sensitivity analysis excluded participants with prevalent coronary heart disease or heart failure.

^||^This sensitivity analysis additionally adjusted for left atrial volume.

^¶^This sensitivity analysis additionally adjusted for the secondary exposure variables.

**Table 4:** Associations between myocardial mechanics and incident ischemic stroke subtypes in the cardiovascular health study.

Exposure variable*	Any ischemic stroke^†^	Cardioembolic stroke^†^	AF-related cardioembolic stroke^‡^
**Primary exposure variable**			
LA reservoir strain	1.14 (1.04–1.25)	1.42 (1.21–1.67)	1.60 (1.32–1.95)
**Secondary exposure variables**			
LV average longitudinal strain	1.23 (1.12–1.34)	1.27 (1.10–1.48)	1.23 (1.03–1.46)
LV early diastolic strain rate	1.23 (1.13–1.35)	1.19 (1.03–1.37)	1.11 (0.94–1.32)
LV septal e’ velocity	1.18 (1.09–1.29)	1.31 (1.14–1.50)	1.28 (1.09–1.52)
LV lateral e’ velocity	1.09 (1.01–1.19)	1.12 (0.98–1.29)	1.08 (0.92–1.27)

LA, left atrial; LV, left ventricular.

*Reported results represent the hazard ratio (95% confidence interval) for ischemic stroke per each standard deviation absolute decrease in strain and decrease in strain rate and e’ velocity. Lower absolute values represent worse strain.

^†^Models were adjusted for age, race, sex, study site, speckle-tracking analyst, chamber-specific echocardiogram image quality, education, body mass index, coronary heart disease, heart failure, diabetes, systolic blood pressure, anti-hypertension medication, high-density lipoprotein level, low-density lipoprotein level, log-transformed triglyceride level, smoking status, LV ejection fraction, and atrial fibrillation as a time-varying covariate.

^‡^Models were adjusted as in footnote b except for atrial fibrillation as a time-varying covariate.

## Discussion

In a community-based, longitudinal cohort study, we found that echocardiographic evidence of myocardial mechanical dysfunction was associated with subsequent ischemic stroke. Associations between myocardial strain indices and stroke were not substantially attenuated after adjustment for vascular risk factors, left ventricular ejection fraction, left atrial volume, and clinically apparent AF. These associations persisted after excluding participants with clinically apparent coronary heart disease or heart failure. Left atrial mechanics and left ventricular mechanics remained associated with ischemic stroke independently of each other.

Previous studies have examined associations between echocardiographic measures of myocardial strain and incident AF^14–16^, markers of left atrial thromboembolism^18,19^, and stroke in patients with AF^20,21^ or acute MI^33^. Several studies have examined the association between myocardial strain and a composite of major adverse cardiovascular endpoints, but these involved single-center cohorts^34,35^ or lacked power to assess ischemic stroke specifically^36,37^. Single-center case–control studies found worse left atrial reservoir strain in patients with cardioembolic^38^ or cryptogenic stroke compared to controls^39,40^. An analysis of data from a prospective, longitudinal cohort study found a non-significant association between left atrial global longitudinal strain on cardiac magnetic resonance imaging and cerebrovascular events^41^. In this context, our study provides novel findings that early myocardial mechanical dysfunction, including in the absence of clinically overt coronary heart disease or heart failure, is associated with subsequent ischemic stroke in the general population.

Our finding of an association between early left ventricular dysfunction and ischemic stroke, independent of left atrial dysfunction and of similar magnitude with both cardioembolic and non-cardioembolic stroke subtypes, suggests that measurement of cardiac mechanics may allow detection of early end-organ damage from systemic microvascular and microvascular disease affecting both the heart and brain. Furthermore, our findings may have implications for the evolving understanding of the substrate necessary for cardiac embolism. Common cardiac conditions that have long been established as cardioembolic sources, such as clinically apparent AF and clinically apparent MI, may be phenotypically broader than previously recognized. Isolated episodes of subclinical AF^42^ and markers of left atrial remodeling^43,44^ have been associated with a higher risk of ischemic stroke independent of clinically apparent AF. Beyond the acute period of stroke risk traditionally associated with clinically apparent MI, chronic myocardial scar from temporally remote or silent MI may also be a risk factor for cardiac embolism^45,46^. Similarly, while gross structural abnormalities such as chamber dilatation^47,48^ and frank cardiac mechanical dysfunction, as manifested by reduced ejection fraction^41,49^ and emptying velocity^50^, have long been recognized as sources of thromboembolism, our results raise the possibility that cardiac embolism might occur even earlier along the spectrum of myocardial dysfunction. Such a possibility is supported by the common occurrence of strokes that lack a cardioembolic source per traditional criteria but nevertheless appear to have arisen from a central embolic source^9^. At a minimum, our findings imply that the currently recognized substrate for cardiac embolism is heralded by more subtle evidence of cardiac dysfunction. Although a single echocardiographic assessment is unlikely to provide robust risk prediction for outcomes many years later, our findings suggest opportunities to test whether serial cardiac assessments, in the form of heart-rhythm monitoring, biomarker assays, echocardiography, and other diagnostic modalities, can identify cardioembolic risk earlier than current practice, in which many ischemic stroke patients present with previously undiagnosed cardioembolic sources such as AF^51^ that may not have resulted in stroke if recognized and treated earlier. Our findings also imply opportunities to test whether stroke risk can be reduced by early recognition of myocardial dysfunction and institution of intensive upstream therapies to arrest or reverse its progression^52–54^, as well as early use of tailored antithrombotic therapy in high-risk patients in sinus rhythm^55^, in addition to the current approach of managing downstream complications such as AF with anticoagulation.

Our study has several limitations. First, participants did not undergo continuous heart-rhythm monitoring or systematic serial echocardiographic assessments throughout follow-up. Therefore, we could not examine the degree to which associations between indices of cardiac mechanics and stroke were mediated by interim development of subclinical AF or grossly abnormal systolic function. Second, participants with available speckle-tracking echocardiogram data differed from the overall CHS cohort, especially in regard to race. There were fewer African-American participants in the study population analyzed than in the overall CHS cohort, and future studies should strive to include more African-American individuals and other understudied populations in studies of myocardial strain and stroke risk. Third, myocardial strain was measured from digitized versions of analog echocardiogram images. This method substantially differs from contemporary clinical and research protocols involving direct digital acquisition and strain measurement, most notably in regards to a slower frame rate than currently used. On the other hand, our approach enabled more prolonged longitudinal follow-up for outcomes than would be possible with more contemporary cardiac imaging protocols. In population-based studies, our methodology has been useful in showing associations of cardiac strain measures with disease phenotypes and prediction of outcomes, supporting the validity of our methods to identify important relationships between myocardial function and disease that can be expected to apply to contemporary patients^13,56^. Moreover, any imprecision of our methods would have decreased our ability to find meaningful associations, suggesting that our findings may actually understate the association of cardiac strain measures with incident ischemic stroke. Fourth, given the long follow-up period, there was a substantial rate of mortality in the study population. This may affect calculations of cumulative rates of stroke, although it should not affect our estimates of cause-specific HRs^57^.

Based on our findings, it appears that subtle cardiac dysfunction that can ultimately lead to stroke may be identifiable at an early stage. These findings highlight an opportunity to identify cardioembolic risk earlier and institute preventive measures including upstream therapeutic strategies directed at preventing further progression of thrombogenic cardiac dysfunction. Such a strategy may be a promising avenue for reducing the burden of cardioembolic stroke, a severe and increasingly common form of a disease that is the second-leading cause of death^1^ worldwide.

## Supplementary Information

Supplementary Information.

## Data Availability

The data used for this study is available upon reasonable request to the corresponding author.
