# Supplementary material for: Cardiac mechanics and incident ischemic stroke: the Cardiovascular Health Study
Source: Sci Rep. 2021 Aug 30;11:17358. doi: 10.1038/s41598-021-96702-z (PMC8405795; doi:10.1038/s41598-021-96702-z)
Supplement: Supplementary file 1 — Supplementary Information. [file 41598_2021_96702_MOESM1_ESM.docx]

**SUPPLEMENTAL MATERIAL**

**Cardiac Mechanics and Incident Ischemic Stroke in the Cardiovascular Health Study**

Hooman Kamel, MD, MS^1,2^; Traci M. Bartz, MS^3^; W. T. Longstreth, Jr., MD, MPH^4,5,6^; Mitchell S.V. Elkind, MD, MS^7,8^; John Gottdiener, MD^9^; Jorge R. Kizer, MD, MSc^10^; Julius Gardin, MD, MBA^11^; Jiwon Kim, MD^12^; Sanjiv Shah, MD^13^

^1^Clinical and Translational Neuroscience Unit, Department of Neurology and Feil Family Brain and Mind Research Institute, ^2^Division of Neurocritical Care, and ^12^Division of Cardiology, Weill Cornell Medicine, New York, NY; Departments of ^3^Biostatistics, ^4^Neurology, ^5^Medicine, and ^6^Epidemiology, University of Washington, Seattle, WA; ^7^Department of Neurology, Vagelos College of Physicians and Surgeons, and ^8^Department of Epidemiology, Mailman School of Public Health, Columbia University, New York, NY; ^9^Division of Cardiology, University of Maryland, Baltimore, MD; ^10^Cardiology Section, San Francisco Veterans Affairs Health Care System, and Departments of Medicine, Epidemiology, and Biostatistics, University of California San Francisco, San Francisco, CA; ^11^Division of Cardiology, Rutgers New Jersey Medical School, Newark, NJ; ^13^Division of Cardiology, Northwestern University Feinberg School of Medicine, Chicago, IL

**Table of Contents**

Supplemental Figure I Page 2

Supplemental Table I Page 3

**Supplemental Figure I.** Flow Diagram of Cardiovascular Health Study Participants Included in Analysis of Cardiac Mechanics and Incident Ischemic Stroke.

**
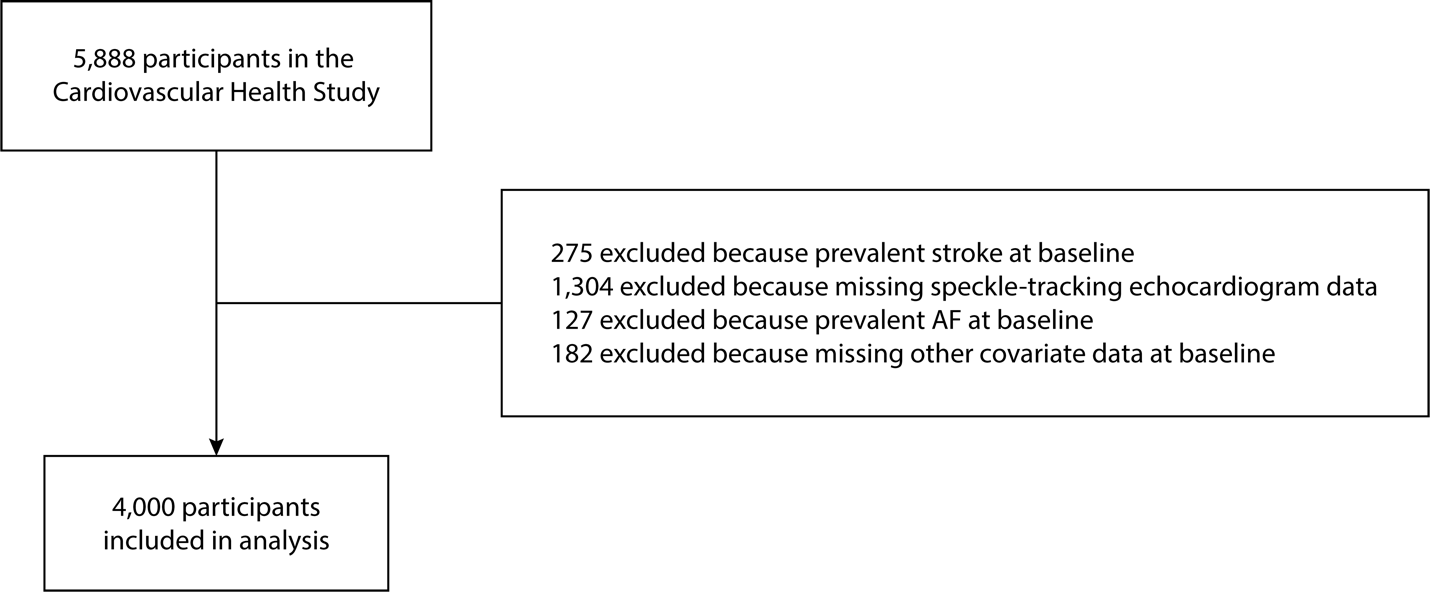
**

| **Supplemental Table I. Intra- and Inter-observer Variability of Indices of Cardiac Mechanics in the Cardiovascular Health Study.** | | | | |
| --- | --- | --- | --- | --- |
|  | **Mean ± SD** | **ICC (95% CI)** | **Mean Bias (95% CI)** | **CV** |
| **Intraobserver variability (n=46)** | | | | |
| LV longitudinal systolic strain, % | 13.9 ± 4.06 | 0.98 (0.96-0.99) | 0.05 (-0.31, 0.41) | 7.0% |
| LV early diastolic strain rate, s^-1^ | 0.64 ± 0.26 | 0.95 (0.92-0.97) | -0.02 (-0.005, 0.02) | 16.1% |
| LA reservoir strain, % | 42.9 ± 14.9 | 0.99 (0.98-0.99) | 0.05 (-0.72, 0.82) | 4.6% |
| **Interobserver variability (n=96)** | | | | |
| LV longitudinal systolic strain, % | 14.2 ± 4.0 | 0.94 (0.91-0.96) | -0.58 (-0.97, -0.20) | 9.8% |
| LV early diastolic strain rate, s^-1^ | 0.64 ± 0.22 | 0.90 (0.84-0.93) | -0.04 (-0.07, -0.02) | 16.1% |
| LA reservoir strain, % | 40.4 ± 16.9 | 0.96 (0.94-0.97) | 0.56 (-0.71, 1.84) | 11.5% |
| Abbreviations: LV, left ventricular; LA, left atrial; ICC, intraclass correlation; CV, coefficient of variation. | | | | |
